# SIRPB1 regulates inflammatory factor expression in the glioma microenvironment via SYK: functional and bioinformatics insights

**DOI:** 10.1186/s12967-024-05149-z

**Published:** 2024-04-09

**Authors:** Ren Geng, Yao Zhao, Wanzhen Xu, Xiaoshan Ma, Yining Jiang, Xuefei Han, Liyan Zhao, Yunqian Li

**Affiliations:** 1 Department of Neurosurgery, First Hospital of Jilin University, No. 1, Xinmin Street, Chaoyang District, Changchun, China; 2 Department of Neurosurgery, Qilu Hospital of Shandong University Dezhou Hospital, Dezhou, China; 3 Department of Blood Transfusion, Second Hospital of Jilin University, No. 4026, Yatai Street, Nanguan District, Changchun, China

**Keywords:** SIRPB1, Glioma, Biomarker, Tumor microenvironment, Prognosis

## Abstract

**Background:**

SIRPB1 expression is upregulated in various tumor types, including gliomas, and is known to contribute to tumor progression; nevertheless, its function in the immune milieu of gliomas is still mainly unknown.

**Methods:**

This study, we analyzed 1152 normal samples from the GTEx database and 670 glioma samples from the TCGA database to investigate the relationship between the expression of SIRPB1 and clinicopathological features. Moreover, SIRPB1 gene knockout THP-1 cell lines were constructed using CRISPR/Cas9 and were induced into a co-culture of macrophages and glioma cells in vitro to learn more about the role of SIRPB1 in the glioma immune milieu. Lastly, we established a prognostic model to predict the effect of SIRPB1 on prognosis.

**Results:**

Significantly higher levels of SIRPB1 expression were found in gliomas, which had an adverse effect on the immune milieu and correlated poorly with patient survival. SIRPB1 activation with certain antibodies results in SYK phosphorylation and the subsequent activation of calcium, MAPK, and NF-κB signaling pathways. This phenomenon is primarily observed in myeloid-derived cells as opposed to glioma cells. In vitro co-culture demonstrated that macrophages with SIRPB1 knockout showed decreased IL1RA, CCL2, and IL-8, which were recovered upon ectopic expression of SIRPB1 but reduced again following treatment with SYK inhibitor GS9973. Critically, a lower overall survival rate was linked to increased SIRPB1 expression. Making use of SIRPB1 expression along with additional clinicopathological variables, we established a nomogram that showed a high degree of prediction accuracy.

**Conclusions:**

Our study demonstrates that glioma cells can be activated by macrophages via SIRPB1, subsequently reprogramming the TME, suggesting that SIRPB1 could serve as a promising therapeutic target for gliomas.

**Supplementary Information:**

The online version contains supplementary material available at 10.1186/s12967-024-05149-z.

## Background

Gliomas provide a serious challenge to neuro-oncology because of their aggressive nature and dismal prognosis, which are characteristics of their high malignancy [1]. Gliomas have an especially complex immune microenvironment, which is made up of many immune cells and pro-tumorigenic cytokines that aid in the development of tumors and immune evasion [2]. Although the brain has been proven to be privileged to a certain extent, subsequent studies have shown that the brain does not completely rule out immune cells from peripheral blood [3]. The majority of the immune subset in glioblastoma is made up of myeloid-derived cells, which include neutrophils, dendritic cells (DC), microglia, tumor-associated macrophages (TAM), and bone marrow-derived suppressor cells (MDSC). TAM secretes chemokines and substances that promote the growth and survival of tumor cells, making up the majority of the non-tumor cells in gliomas [4, 5].

Signal Regulatory Protein Beta 1 (SIRPB1) is a notable molecule within this complex environment. SIRPB1, a cell surface glycoprotein, is mainly expressed in monocytes and dendritic cells. It is a member of the immunoglobulin superfamily as well as the signal regulatory protein family [6, 7]. Some studies indicate that DAP12 expression is crucial for surface presentation of SIRPB1 [8]. Although SIRPB1’s functions were initially identified outside of oncological settings, new research highlights the critical role it plays in the glioma immunological landscape, especially when it comes to spleen tyrosine kinase (SYK) [9]. SYK significantly impacts the tumor microenvironment and antitumor immunity efficacy [10], influencing glioma progression by altering cell proliferation and migration pathways [11]. Our knowledge of the significance of SIRPB1 and SYK signaling in cancer biology has significantly changed as a result of this interaction's recognition as being important in glioma immune response modulation.

We attempt to analyze the role of SIRPB1 in the glioma immune microenvironment in detail and determine its activation and related pathways, the release of tumor-promoting cytokines, and its role in prognosis, which provides a basis for further exploration of SIRPB1 as a potential therapeutic target, given the complexity and importance of the glioma immune microenvironment and the incomplete understanding of its mechanism.

## Methods

### RNA sequencing and clinic information from TCGA and GTEx data repository

TCGA and GTEx expression data processed by the TOIL process [12] were obtained from XENA [13]. The prognostic data were obtained from the study of Liu et al. [14]. The clinical data, including WHO grade, IDH mutation status, and 1p/19q co-deletion status, were sourced from the study by Ceccarelli et al. [15]. Samples from GBM and LGG cohorts were included in the analysis of TCGA glioma samples. Missing data were noted, and tumor samples were categorized into high or low SIRPB1 expression groups based on median levels. Our study followed ethical guidelines, including patient consent, and complied with Xena, TCGA and GTEx publication standards.

### Differentially expressed genes (DEGs) analysis

The package DESeq2 was utilized for the analysis of differential gene expression [16]. Criteria for significant gene identification included an absolute log_2_ fold change > 1.5 and an adjusted *P* < 0.01. Results will be visualized through Volcano plots and heatmaps. We further used Toil-processed RNAseq data in TPM format from TCGA and GTEx to compare SIRPB1 expression across 33 tumor types [12].

### Enrichment analysis

For enrichment analysis, we employed the R package ClusterProfiler [17] to conduct gene ontology (GO) enrichment analysis, covering biological process (BP), cellular components (CC), and molecular function (MF) categories [18, 19]. We also performed the Kyoto Encyclopedia of Genes and Genomes (KEGG) pathway enrichment analysis [20–22]. A false discovery rate (FDR) threshold of < 0.05 was used to identify significant GO functions and KEGG pathways.

### Gene set enrichment analysis (GSEA)

In this study, we used the package clusterProfiler to conduct Gene Set Enrichment Analysis (GSEA) between high and low SIRPB1 expression groups [17]. We used C2.cp.v7.0.symbols.gmt[Curated] and H.all.v7.0.symbols.gmt[Hallmarks] as reference gene sets. Significance criteria included an adjusted *P* < 0.05, FDR q-value < 0.25, and an absolute normalized enrichment score (|NES|) > 1.

### Immune infiltration analysis and immunological constant of rejection (ICR) analysis

We used the ssGSEA approach with the package GSVA to assess the infiltration of 24 immune cell types in malignancies [23]. This list includes macrophages, mast cells, neutrophils, eosinophils, various NK and DC subsets, multiple T cell types, and B cells. Using hallmark gene expression profiles for these cells, we calculated relative enrichment scores for each cell type in individual samples.

To identify the cell types most closely linked with SIRPB1, we consulted two single-cell sequencing datasets: one focused on astrocytoma [24] and another on glioblastoma [25]. SIRPB1 expression across different cell types was analyzed using the Single Cell Portal.

The ICR scoring method refers to Roelands et al. [26, 27]. Based on the TPM data of 20 ICR genes (IFNG, IRF1, STAT1, IL12B, TBX21, CD8A, CD8B, CXCL9, CXCL10, CCL5, GZMB, GNLY, PRF1, GZMH, GZMA, CD274, PDCD1, CTLA4, FOXP3 and IDO1), the TCGA-GBMLGG cohort was clustered using R package ConsensusClusterPlus (v.1.66.0) [28]. The optimal number of clusters for the best sample separation is determined to be two based on the Calinski-Harabasz criterion. The samples were classified as ICR-high and ICR-low based on the degree of ICR gene expression.

### Protein–protein interaction (PPI) analysis

To explore interactions among the DEGs identified in Sect. "Differentially Expressed Genes (DEGs) Analysis" of the Methods, we performed a PPI analysis via Metascape [29]. We conducted protein–protein interaction enrichment for the DEGs list using databases like BioGrid [30], InWeb_IM [31], and OmniPath [32]. Hub genes identified through Metascape's MCODE algorithm [33] were further analyzed for enrichment.

### Single-cell data analysis and pseudotime analysis

We used the GSE117891 dataset [34] for single-cell analysis. Cells were annotated and clustered by type. Microglial and macrophage cells were then sorted into high and low IL1RN expression groups for GO, KEGG, and GSEA analyses. The Monocle2 and Monocle3 algorithms were used to do a pseudotime analysis of IL1RN [35–37].

### Statistical analysis

For data analysis, we employed R software (version 3.6.2). Image analysis was done via Fiji, while GraphPad Prism 8.0 was used for quantitative assessments. Statistical comparisons were made using the Student's t-test and the Wilcoxon test. For multiple-group comparisons, we used analysis of variance. Survival analysis was executed using the Kaplan–Meier curve's log-rank test via the survminer package (ver0.4.8, https://cran.r-project.org/web/packages/survminer/index.html).

### Construction and evaluation of the nomogram

To identify survival-related factors, we integrated SIRPB1 expression and key clinicopathological variables into a multivariate COX regression-based nomogram. Utilizing the R package rms (ver.5.1–4, https://cran.r-project.org/web/packages/rms/index.html), we generated nomograms and calibration plots to calculate patient scores. The C-index was used to assess how accurate the nomogram was at making predictions.

### Immunohistochemical staining and immunofluorescence

In summary, paraffin slides from glioma patients were obtained from the First Hospital of Jilin University’s Pathology Department. Slides underwent dewaxing, antigen retrieval, and peroxidase blocking. After BSA blocking, primary and secondary antibodies (detailed in Additional file 1: Table S1) were applied sequentially, followed by DAB chromogenic solution for immunohistochemistry or TSA for multicolor immunofluorescence. Slides were then stained with DAPI, treated for autofluorescence, and scanned.

### Cell lines and culture conditions

Cell lines GL261, U87MG, T98G, U118MG, A172, LN229, and U251MG were sourced from iCell Bioscience Inc., while N9, BV2, LN18, and HEK293T were obtained from Neurosurgery Lab. THP-1 and HMC3 came from Procell Life Science, and RAW264.7 from Cas9X. HMC3, U87MG, and T98G were cultured in MEM with added Sodium Pyruvate. THP-1 cells were cultured in RPMI-1640 with β-mercaptoethanol. The rest cells were cultured in DMEM with 10% FBS and 1% PS. All cells were mycoplasma-tested and STR-verified (for all human-sourced cell lines) and cultured at 37 ℃ in 5% CO2.

### Plasmids, lentivirus packaging

For SIRPB1 targeting, we used pLenti-Crispr v2 with sgRNAs designed for exon 1 (sgRNA1 5ʹ-GAATGCCCGTGCCAGCCTCC-3ʹ, sgRNA2 5ʹ-GGAGGCTGGCACGGGCATTC-3ʹ). A synonymous mutant plasmid was also created, rendering sgRNA1 ineffective. A FLAG peptide sequence was added, and the plasmid was assembled using pTSB-CMV-puro. All plasmid was constructed by Transheep, Shanghai, China.

For lentivirus production, 80%-confluent HEK293T cells in 10cm dishes were switched to OPTI-MEM. Following TransEXP guidelines, we mixed pMD2.G (4 μg), psPAX2.0 (8 μg), and target plasmids (12 μg) in 1.5 ml OPTI-MEM, which was combined with another 1.5 ml OPTI-MEM containing 60 μl of transfection reagents. After a 20 min incubation, the mixture was added to the dish. Culture supernatant was collected at 48 and 72 h, filtered through a 0.45 μm PES filter, and concentrated using 5 × PEG8000. After overnight storage at 4 ℃, it was centrifuged at 4 ℃ and 4000 × *g* for 20 min. The supernatant was discarded, and the lentivirus was resuspended in a serum-free medium.

### Construction of SIRPB1 knockout THP-1 cell lines

THP-1 cells were infected by SIRPB1-WT/KO lentivirus with 8 μg/ml polybrene. After 48 h, 5 μg/ml puromycin was added. Post-7-d culture, T7E1 digestion confirmed gene editing using a Genome-Editing Mutation Detection Kit (Beyotime). Cells were then diluted to 2 cells/ml and cultured in 96-well plates for 21 day. Genomic DNA was extracted using the Animal Tissue DNA Isolation Kit (FOREGENE) and verified via PCR with specific primers. Sanger sequencing at Sangon Biotech identified base deletions.

### Induction and polarization of THP-1 macrophages

For THP-1 cells in logarithmic growth, density was adjusted to 5 × 10^5^/ml and cultured in 6-well plates or 10 mm dishes with 100 ng/ml PMA for 48 h. After washing with PBS, cells were cultured in serum-free RPMI-1640 for 24 h. Depending on experimental needs, inhibitors GS-9973, SC-75741 (Targetmol), or DMSO were added. For M1 polarization, 100 ng/ml LPS (Beyotime) and 20 ng/ml INF-γ (Absin) were added and cultured for 48 h. For M2 polarization, 20 ng/ml IL-4 and 20 ng/ml IL-13 (Absin) were added and cultured for 48 h.

### Protein extraction and western blot

In this study, nuclear and cytoplasmic proteins were separated using a Nuclear and Cytoplasmic Protein Extraction Kit (Beyotime). For total protein, cells were treated with 5 ug/ml Brefeldin A (Beyotime) for 4 h and lysed with RIPA lysate (Beyotime) containing protease and phosphorylase inhibitors (targetmol). SDS-PAGE gel electrophoresis was performed as needed, using gels of varying concentrations (6, 8, 10, 12, 15% from Sangon Biotech.), and specific antibodies (detailed in Additional file 1: Table S1) were applied.

### Co-immunoprecipitation (Co-IP)

After discarding the culture medium, cells were lysed on ice using IP lysate (Beyotime) containing protease inhibitors. The protein extract was incubated overnight at 4 ℃ with either mouse IgG or FLAG antibody. Protein A/G beads (Abmart) were added and incubated at 4 ℃ for 6 h, followed by elution with 1 × loading buffer.

### Cellular calcium detection

For calcium assays, THP-1 cells induced into macrophages were loaded with a Fluo-4 calcium probe using a Fluo-4 Calcium Assay Kit (Beyotime). Cells were then exposed to U87 cell culture supernatant and imaged every 5 s under a fluorescence microscope. Images were analyzed by FIJI software.

### Flow cytometry

The induced THP-1 macrophages were collected, Fc receptors were blocked by human Fc Receptor Blocking Solution (Maokangbio), stained with FITC Anti-Mouse/Human CD11b Antibody (Elabscience, clone:M1/70), 7-AAD (Elabscience), APC Anti-Human CD206 Antibody (Elabscience, clone:15–2) and PE Anti-Human CD86 Antibody (Elabscience, clone:BU63), and detected and analyzed by Beckman cytoflex flow cytometry.

### Real-time quantitative PCR (RT-qPCR)

Total RNA was extracted using TRIGene (Genstar). 1 μg of RNA was reverse transcript using the RT Easy II kit (Foregene). qPCR was performed using SYBR Green Master Mix (Yeasen), with GAPDH as the reference gene. Primer sequences are in Additional file 1: Table S2.

### Luminex multi-cytokines detection

For co-culture experiments, U87 cells were placed in a transwell chamber above THP-1-induced macrophages in a 6-well plate. After 48 h, culture media from both layers were collected, filtered through a 0.45 μm filter, and stored at – 80 ℃. Luminex assays were conducted by Labex (Shanghai). Data were analyzed post-standardization per million THP-1 macrophages.

## Results

### Clinical characteristics

In this study, we analyzed 670 RNA-seq datasets with clinical data from TCGA LGG and GBM projects (Table 1). The cohort consisted of 386 males and 284 females, median age of 46. The data included 216 WHO-II, 237 WHO-III, and 160 WHO-IV grade cases. IDH mutations were present in 424 patients (64.1%) and 1p/19q co-deletion in 168 (25.3%). SIRPB1 expression correlated significantly with WHO grade, IDH status, 1p/19q co-deletion, primary therapy outcome, histological type, and age (all *P* < 0.001) but not with race, PIK3CA status, EGFR status, and gender.

**Table 1:** Characteristics and association between SIRPB1 expression and clinicopathologic features of patients with glioma based on TCGA

Variable	No	SIRPB1 expression		*P*
		Low (%)	High (%)	
WHO grade				
WHO-II	216	138 (46.9%)	78 (24.5%)	< 0.001
WHO-III	237	127 (43.2%)	110 (34.5%)	
WHO-IV	160	29 (9.9%)	131 (41.1%)	
IDH status				
Mutant	424	266 (80.4%)	158 (47.9%)	< 0.001
Wild type	237	65 (19.6%)	172 (52.1%)	
1p/19q co-deletion				
Co-deletion	168	123 (36.8%)	45 (13.6%)	< 0.001
Non-co-deletion	496	211 (63.2%)	285 (86.4%)	
Primary therapy outcome				
CR	135	93 (35.1%)	42 (23.5%)	0.016
PD	103	50 (18.9%)	53 (29.6%)	
PR	62	35 (13.2%)	27 (15.1%)	
SD	144	87 (32.8%)	57 (31.8%)	
Gender				
Female	284	154 (46.0%)	130 (38.8%)	0.072
Male	386	181 (54.0%)	205 (61.2%)	
Race				
Asian	13	6 (1.8%)	7 (2.1%)	0.897
Black or African American	32	15 (4.6%)	17 (5.2%)	
White	613	308 (93.6%)	305 (92.7%)	
Histological type				
Astrocytoma	192	103 (30.7%)	89 (26.6%)	< 0.001
Glioblastoma	160	29 (8.7%)	131 (39.1%)	
Oligoastrocytoma	128	75 (22.4%)	53 (15.8%)	
Oligodendroglioma	190	128 (38.2%)	62 (18.5%)	
EGFR status				
Mutant	73	28 (8.6%)	45 (13.7%)	0.05
Wild type	583	299 (91.4%)	284 (86.3%)	
PIK3CA status				
Mutant	49	24 (7.3%)	25 (7.6%)	1
Wild type	607	303 (92.7%)	304 (92.4%)	
Age^#^		42.00 [33.00,55.00]	50.00 [36.00,61.00]	< 0.001

^#^Shown as median [IQR], Wilcoxon rank sum test

*CR* complete response, *PD* progressive disease, *PR* partial response, *SD* stable disease

### Differential expression of SIRPB1

**Fig. 1:**
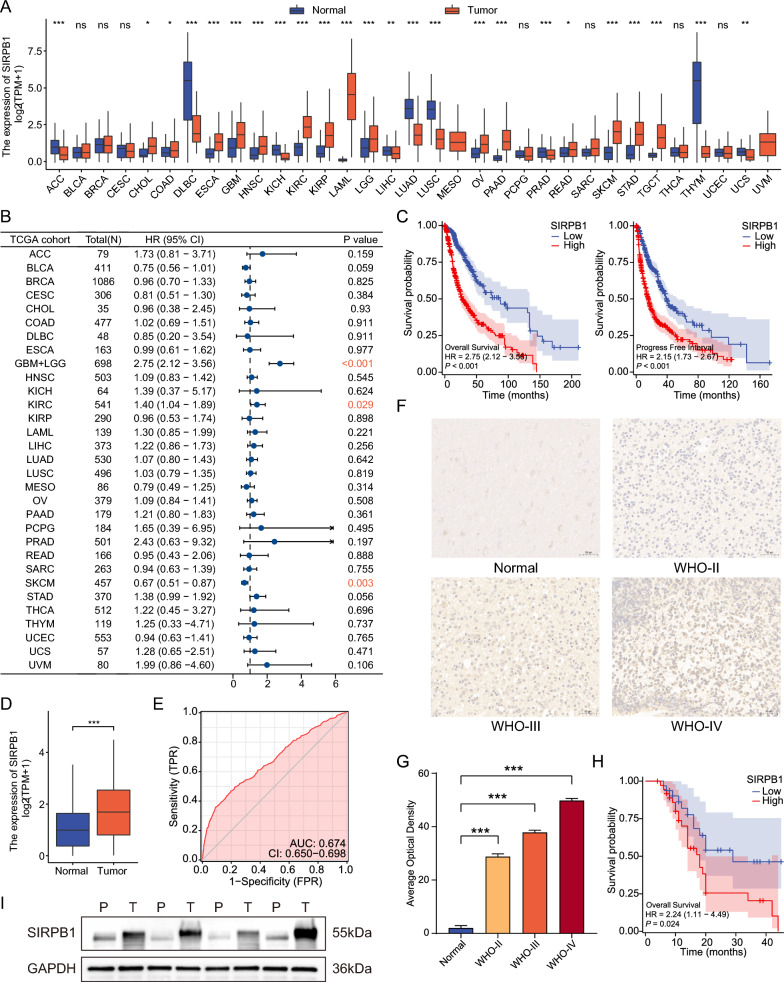
**A** SIRPB1 expression in 33 tumor types vs. controls; Wilcoxon test. **B** Forest plot of SIRPB1's prognostic value in TCGA tumor types. **C** Kaplan–Meier curves for OS and PFI. **D** SIRPB1 levels in normal vs. glioma tissues; Wilcoxon test. **E** ROC curve for SIRPB1's diagnostic accuracy in gliomas; x-axis: FPR, y-axis: TPR. **F** Immunohistochemical staining of SIRPB1 in normal and glioma brain tissues and (**G**) statistical analysis. **H** Kaplan–Meier curves for OS of glioma patients in First Hospital of Jilin University. **I** Western blot of SIRPB1 in paracancerous (**P**) and glioma (**T**) brain tissues. Significance: ns: *P* ≥ 0.05, *P < 0.05, **P < 0.01, ***P < 0.001

We compared the expression of SIRPB1 in normal GTEx samples with 33 TCGA cancer types using the Wilcoxon test (Fig. 1A). Elevated SIRPB1 levels were observed in several cancers, including GBM and LGG, while decreased levels were found in cancers like LUAD and PRAD (all *P* < 0.001). The high expression of SIRPB1 is associated with poor prognosis in glioma and kidney renal clear cell carcinoma, but it seems to be a protective factor in skin cutaneous melanoma (Fig. 1B). Kaplan–Meier curves in Fig. 1C show that higher SIRPB1 expression is linked to poorer Overall Survival (OS) and Progression-Free Interval (PFI) in gliomas. SIRPB1 was notably high in glioma samples (*P* < 0.001, Fig. 1D). The ROC curve suggested SIRPB1’s potential as a diagnostic marker (AUC = 0.674, Fig. 1E). In the areas where glioma cells gathered, SIRPB1 staining increased with the increase of glioma grade (Fig. 1F, G). Through the follow-up of 70 patients with different grades of gliomas in this study center, we found that the expression of SIRPB1 was associated with poor prognosis (Fig. 1H). In the fresh glioma tissue samples we collected, the expression of SIRPB1 detected by Western blot was significantly higher than that in paracancerous tissues (Fig. 1I, Additional file 1: Figure S1A).

### The correlation between SIPRB1 expression and immune infiltration

**Fig. 2:**
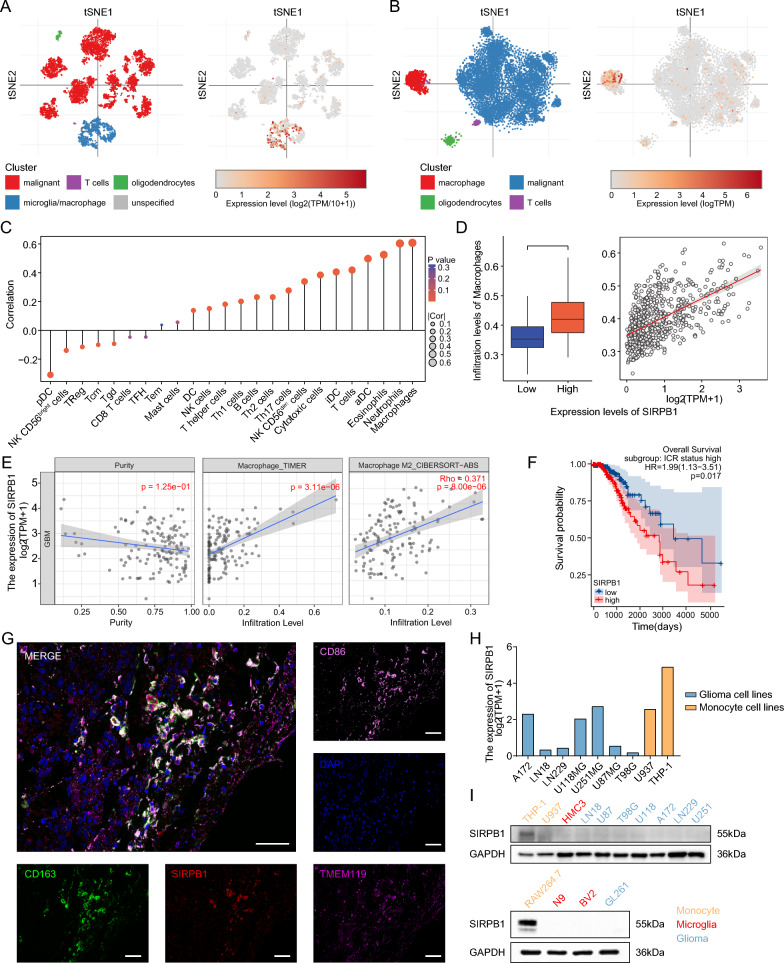
**A**, **B** SIRPB1 expression in astrocytoma and glioblastoma cell clusters. **C** Correlation of SIRPB1 with immune cell infiltration. **D** Association between macrophage infiltration and SIRPB1; Wilcoxon and Spearman tests. **E** SIRPB1 and macrophage correlation in TCGA-GBM via TIMER2.0. **F** Kaplan–Meier curves for OS of TCGA-GBMLGG with ICR-high. **G** Co-localization of SIRPB1 with TMEM119, CD86, and CD163 in glioma. **H** SIRPB1 levels in glioma and monocyte lines from CCLE. **I** SIRPB1 expression in human and mouse glioma, microglia, and monocyte lines

We used the Single Cell Portal to evaluate datasets for glioblastoma and astrocytoma in order to identify the cells in gliomas that had high expression of SIRPB1. SIRPB1 was predominantly found in macrophages or microglia, with low expression in tumor cells (Fig. 2A, B) consistent with our Western blot and immunohistochemical findings.

Next, we assessed immune infiltration in the TCGA database (Fig. 2C). SIRPB1 expression positively correlated with macrophages, neutrophils, and aDCs but negatively with pDCs, Tcm, and Tgd. No significant correlation was found with CD56bright NK cells, dendritic cells, mast cells, and Tem (*P* > 0.05). Wilcoxon and Spearman analyses revealed higher macrophage (*P* < 0.001, cor = 0.574, Fig. 2D) and Th17 cell (*P* < 0.001, cor = 0.297) infiltration in the high-SIRPB1 group, while pDCs, Tcm, and Tgd showed lower levels (*P* < 0.001, cor = − 0.314; *P* = 0.003, cor = − 0.144; *P* = 0.012, cor = − 0.134, respectively; not shown). Further analysis using Timer2.0 confirmed a strong correlation between SIRPB1 and macrophage infiltration in GBM, particularly M2 macrophages (*P* < 0.001, Rho = 0.386 and Rho = 0.371, Fig. 2E). Furthermore, a significant correlation was found between high SIRPB1 levels and unfavorable patient outcomes in samples with high ICR scores (Fig. 2F).

We used the CCLE database to analyze SIRPB1 expression in monocyte and common glioma cell lines in order to validate our hypothesis. THP-1 monocytes showed significantly higher SIRPB1 levels than glioma cell lines (Fig. 2H). Subsequent cultures of glioma cell lines, macrophages, monocytes, and microglial cells in both humans and mice confirmed that SIRPB1 is primarily expressed in macrophages and monocytes (Fig. 2I).

Polychromatic immunofluorescence revealed that SIRPB1 co-localizes with TMEM119 (microglia marker), CD86 (M1 macrophage marker), and CD163 (M2 macrophage marker) indicating that SIRPB1 is predominantly found in TAMs, which in glioma tissues exhibit both M1 and M2 characteristics (Fig. 2G).

### DEG identification and functional enrichment analysis of DEGs

**Fig. 3:**
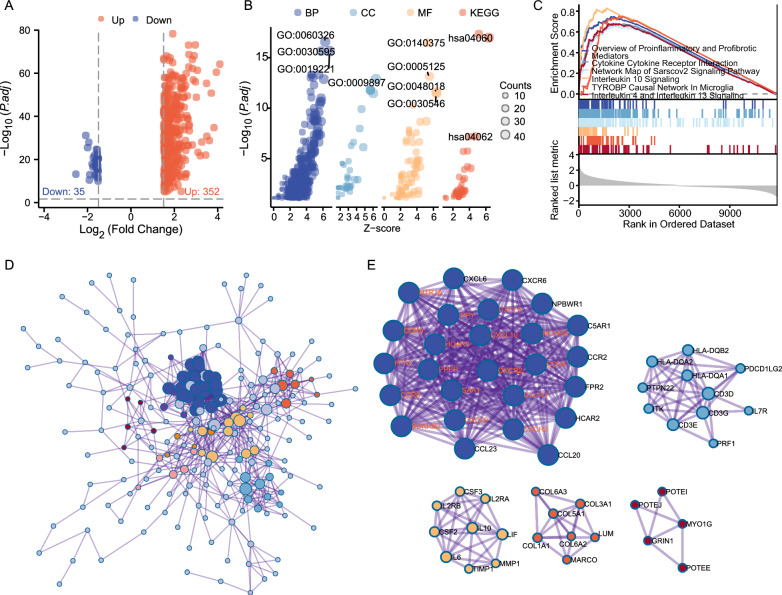
DEGs (TCGA-GBMLGG) Analysis and Functional Enrichment. **A** Volcano plot of DEGs in high- vs. low-SIRPB1 samples. **B** GO and KEGG enrichment terms. **C** GSEA plots. **D** PPI network of DEGs. **E** Key gene modules via MCODE

In the combined TCGA-GBM and LGG datasets, 387 DEGs were identified between high and low SIRPB1 expression samples, based on cut-off criteria of adjust *P* < 0.01 and |log2FC|> 1.5, including 352 up-regulated and 35 down-regulated genes (Additional file 1: Table S3). These DEGs are visualized in a volcano plot (Fig. 3A).

We performed GO and KEGG enrichment analysis on the 387 DEGs to investigate the functional role of SIRPB1 in glioblastoma. A total of 703 significant terms were enriched (Additional file 1: Table S4). According to z-score enrichment results, SIRPB1 is likely involved in cytokine-mediated signaling, cell chemotaxis, and leukocyte migration. The DEGs primarily relate to the external side of the plasma membrane and receptor-ligand activity (Fig. 3B). The enrichment analysis suggests that these DEGs are mainly associated with immune response.

### SIRPB1-related signaling pathways based on GSEA

We used GSEA on low- and high-SIRPB1 samples to identify pathways that are differentially active in gliomas. Significant differences were found in the TYROBP causal network in microglia, IL10 signaling, and cytokine-cytokine receptor interaction (|NES|> 1, adjust *P* < 0.05, FDR q < 0.25, Fig. 3C).

### Protein–protein interaction analysis

To further elucidate SIRPB1's role in glioma, we created a PPI network with 246 nodes and 931 edges using Metascape (Fig. 3D). MCODE analysis identified eleven modules (Fig. 3E). Modules 1–5 were rich in cytokines (e.g., IL6, IL10), cytokine receptors (e.g., CCR2, CCR4), and immune cell markers (e.g., CD3D, CD3E), indicating that the DEGs primarily regulate the immune microenvironment rather than tumor malignancy traits like proliferation and invasion.

### SIRPB1 gene knockout and classical M1 and M2 polarization of macrophages

**Fig. 4:**
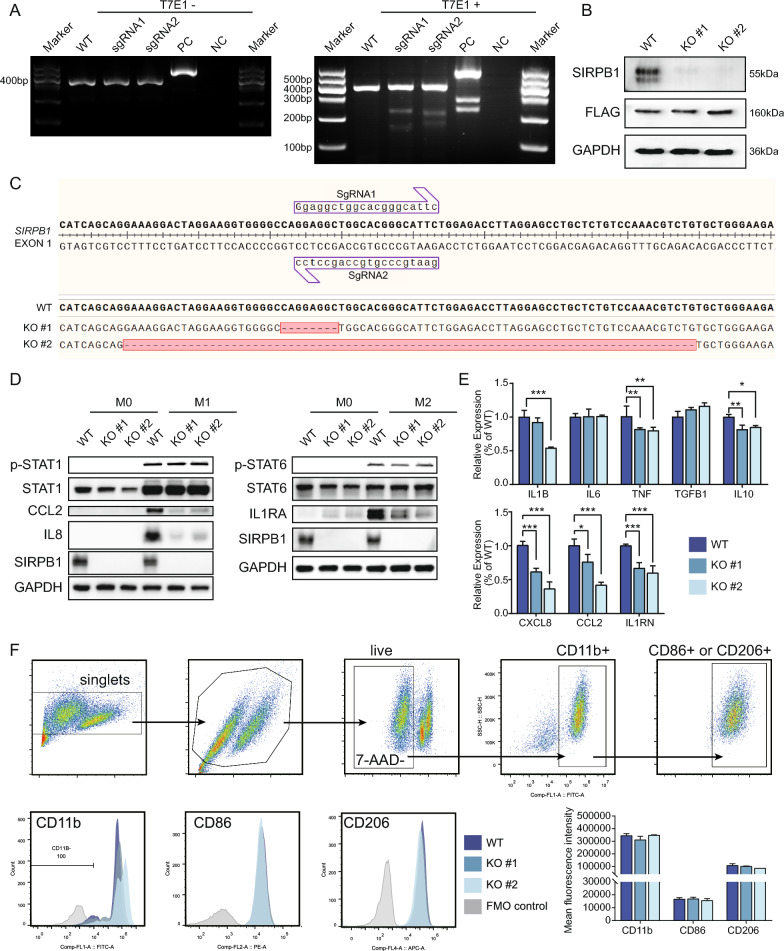
SIRPB1 Knockout and Macrophage Polarization. **A** T7E1 assay results. *WT* wild-type, *NC* negative control, *PC* positive control. **B** SIRPB1 and FLAG-cas9 expression in THP-1 lines. **C** Sanger sequencing of SIRPB1^WT^ and SIRPB1^KO^. **D** Protein expression post-M1/M2 treatments. **E** mRNA levels of M1/M2 markers (*P < 0.05, **P < 0.01, ***P < 0.001, Dunnett’s test). **F** Flow cytometry of CD11b, CD86, CD206 in THP-1 lines

We generated SIRPB1 knockout THP-1 cell lines using CRISPR-Cas9. T7E1 assay confirmed successful DNA mismatch induction by both sgRNAs (Fig. 4A). Sanger sequencing identified clone #1 with 8-base deletions and clone #2 with 80-base deletions, causing frameshift mutations (Fig. 4C). Western blot verified SIRPB1 knockout in both clones (Fig. 4B).

To assess the impact of SIRPB1 knockout on THP-1 cell polarization, we examined STAT1 and STAT6 phosphorylation in M1 and M2 polarizations. No significant changes were observed in the SIRPB1^KO^ group compared to SIRPB1^WT^, suggesting SIRPB1 knockout does not affect STAT phosphorylation (Fig. 4D, Additional file 1: Figure S1B). qPCR revealed that SIRPB1 knockout reduced the transcription levels of IL1B (only for KO#2), TNF, and IL10 but didn’t affect IL6 and TGFB1. Notably, SIRPB1 knockout significantly downregulated IL-8 and CCL2 in M1 and IL1RA in M2 polarization (Fig. 4E). Flow cytometry showed no impact on classical M0, M1, and M2 macrophage surface markers CD11b, CD86, and CD206 (Fig. 4F).

### Investigating the effect of SIRPB1 on IL1RA expression at the single-cell level

**Fig. 5:**
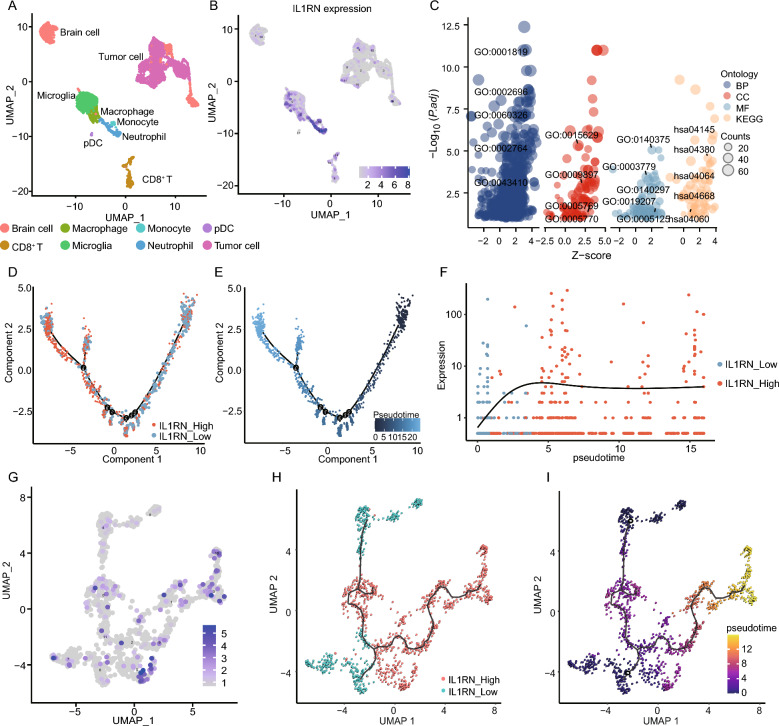
Single-Cell Analysis of IL1RN Expression. **A** UMAP cell clustering. **B** IL1RN expression in all cells. **C** GO and KEGG enrichment for IL1RN. **D**, **E** Cell type and pseudotime trajectory of IL1RN in microglia and macrophages. **F** IL1RN expression dynamics across pseudotime. **G**, **I** UMAP dot plots of IL1RN in microglia and macrophages

We examined a single-cell dataset (GSE117891) that was centered on macrophages and microglia in order to clarify the signaling pathways associated with SIRPB1. We identified 8 cell clusters based on marker genes (Fig. 5A). IL1RN and CXCL8 were highly expressed in neutrophils, while CCL2 was predominant in macrophages-microglia (Fig. 5B and Additional file 1: Figure S2A). In these cells, high IL1RN expression was associated with processes like tumor necrosis factor response and cell chemotaxis (Fig. 5C). KEGG analysis revealed enrichment in pathways like phagosome and TNF signaling (Additional file 1: Table S5). Monocle pseudotime showed a developmental trajectory of increasing IL1RN expression (Fig. 5D-I).

### Co-culturing U87 with macrophages reveals that SIRPB1 can influence the immune microenvironment of gliomas

**Fig. 6:**
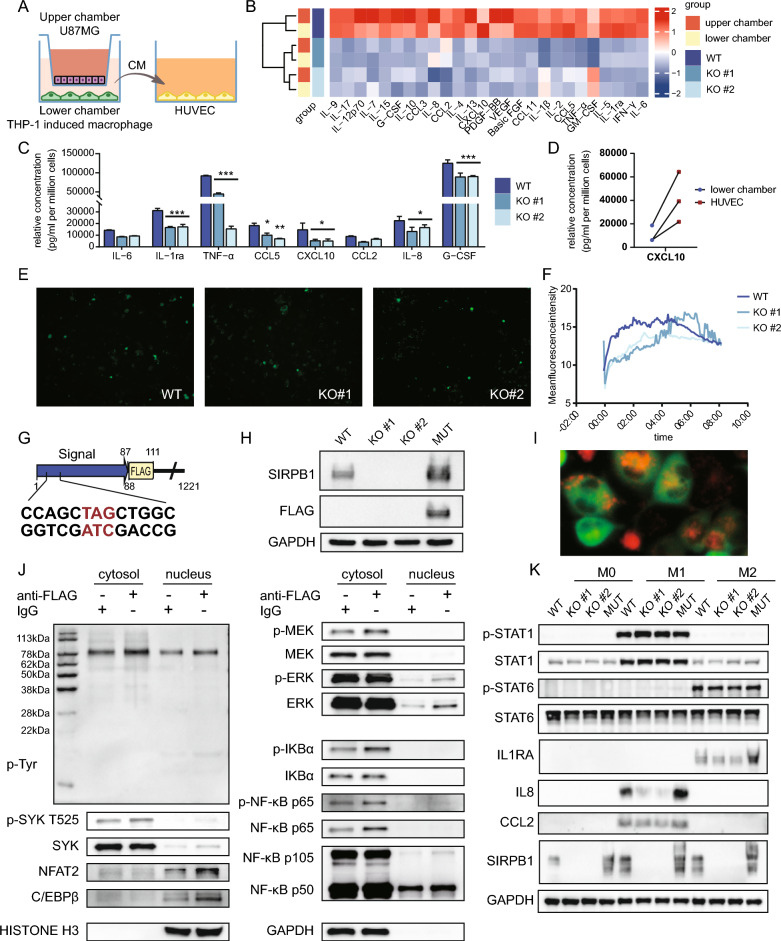
SIRPB1 in Glioma-Macrophage Co-Culture. **A** Transwell setup. **B**, **C** Inflammatory cytokines via Luminex. **D** CXCL10 before and after HUVEC culture. **E**, **F** Fluo-4 staining in THP-1 cells. **G** SIRPB1^MUT^ design. **H** SIRPB1 recovery verified by Western blot. **I** SIRPB1^MUT−GFP^ activation with anti-FLAG. **J** MAPK, NF-κB activation post SIRPB1 activation. **K** CCL2, IL1RA, IL-8 changes in M1/M2 polarization after SIRPB1 recovery

We set up a co-culture system (Fig. 6A) and collected a conditioned medium after 48 h. The medium revealed elevated VEGF, PDGF-BB, IL-8, and G-CSF levels in the upper chamber (U87MG) and higher CCL2, IL1B, and CCL5 levels in the lower chamber (macrophages). SIRPB1^KO^ had lower inflammatory factors than SIRPB1^WT^ (Fig. 6B). Specifically, IL-8 and IL1RA were significantly lower in the SIRPB1^KO^ group (Fig. 6C). After 48 h of HUVEC conditioning, CXCL10 levels surged, but the increase was less pronounced in the SIRPB1^KO^ group (Fig. 6D). We investigated the function of SIRPB1 in calcium signaling and discovered that it mostly affects early-stage calcium signaling, having little effect on the peak but delaying the beginning (Fig. 6E, F).

For recovery experiments, we chose SIRPB1^KO^ #2 and counteracted sgRNA2 effects with synonymous mutations, appending a FLAG peptide (Fig. 6G, H). Post-activation probing revealed increased SYK phosphorylation at 6 h and activated MAPK and NF-kB pathways, evidenced by ERK phosphorylation and NF-κB p50 nuclear translocation. Elevated NFAT2 and C/EBPβ levels were also noted, supporting our earlier findings (Fig. 6I, J, Additional file 1: Figure S1C). Restoring SIRPB1 expression led to recovered IL1RA, CCL2, and IL-8 levels, while STAT1 and STAT6 phosphorylation remained unchanged (Fig. 6K, Additional file 1: Figure S1D).

### Phosphorylation of SYK is necessary for SIRPB1 to reshape immune microenvironment

**Fig. 7:**
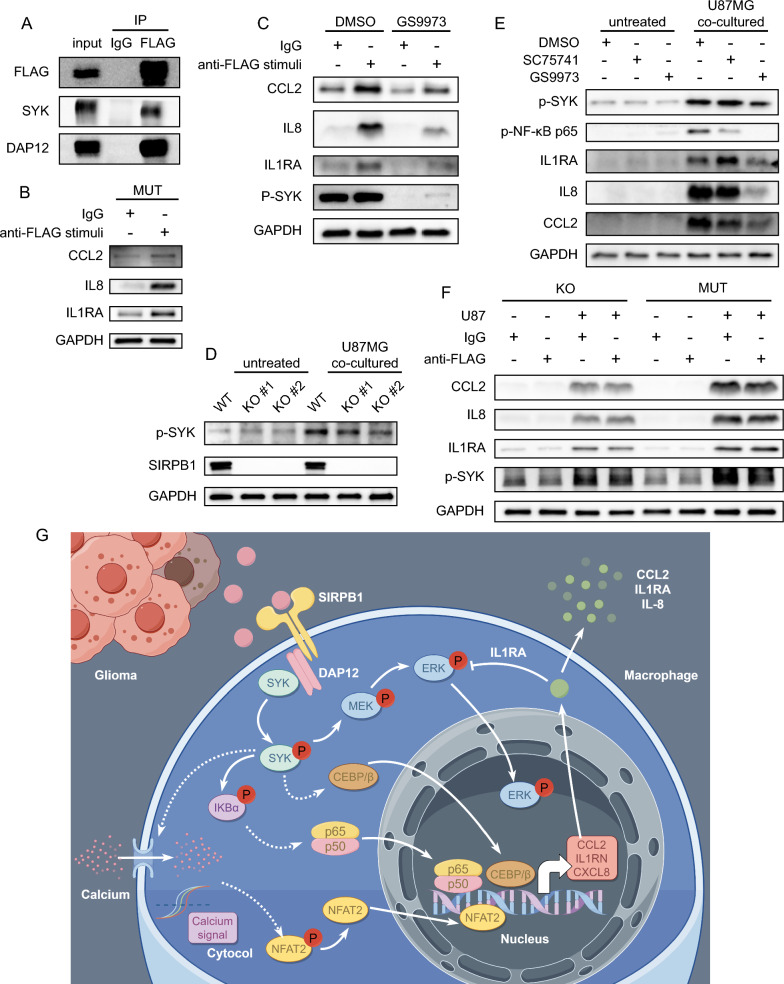
SIRPB1 and SYK in Glioma-Macrophage Interaction. **A** Co-IP analysis of SIRPB1/DAP12/SYK. **B** CCL2, IL-8, IL1RA levels post SIRPB1 activation. **C** SYK inhibitor effects on FLAG antibody stimulation. **D** Impact of SIRPB1 deletion on SYK phosphorylation in glioma-macrophage co-culture. **E** Expression of CCL2, IL-8, IL1RA with NF-κB or SYK inhibitors in co-culture. **F** CCL2, IL-8, IL1RA levels in U87 co-culture post FLAG antibody competition. **G** Schematic diagram of the action mechanism of SIRPB1

We detected that SIRPB1 binds to SYK through DAP12 adaptor protein in macrophages by co-immunoprecipitation (Fig. 7A). Anti-FLAG antibody was used to activate SIRPB1 to up-regulate the expression of CCL2, IL-8 and IL1RA (Fig. 7B, Additional file 1: Figure S1E), which could be blocked by SYK inhibitor GS-9973 (Fig. 7C, Additional file 1: Figure S1F), confirming that SYK was involved. Furthermore, we found that there was a constitutive phosphorylation of SYK in the co-culture experiment. The level of SYK phosphorylation in SIRPB1^KO^ was lower than that in SIRPB1^WT^ at the early stage of co-culture, indicating that SIRPB1 was partially involved in the phosphorylation of SYK. The absence of SIRPB1 could not completely block the phosphorylation of SYK (Fig. 7D, Additional file 1: Figure S1G). We found that compared with 2 μM GS-9973, 5 μM SC-75741 could only slightly inhibit the expression of CCL2 but had no significant inhibitory effect on the expression of IL1RA and IL-8 (Fig. 7E, Additional file 1: Figure S1H). Blocking SIRPB1 binding (without secondary antibody) did not significantly inhibit these expressions (Fig. 7F, Additional file 1: Figure S1I). Combined with the existing results and literature, we can preliminarily explain the role of SIRPB1 in glioma-associated macrophages (Fig. 7G).

### Correlations between SIRPB1 expression and clinical characteristics in glioma patients

**Fig. 8:**
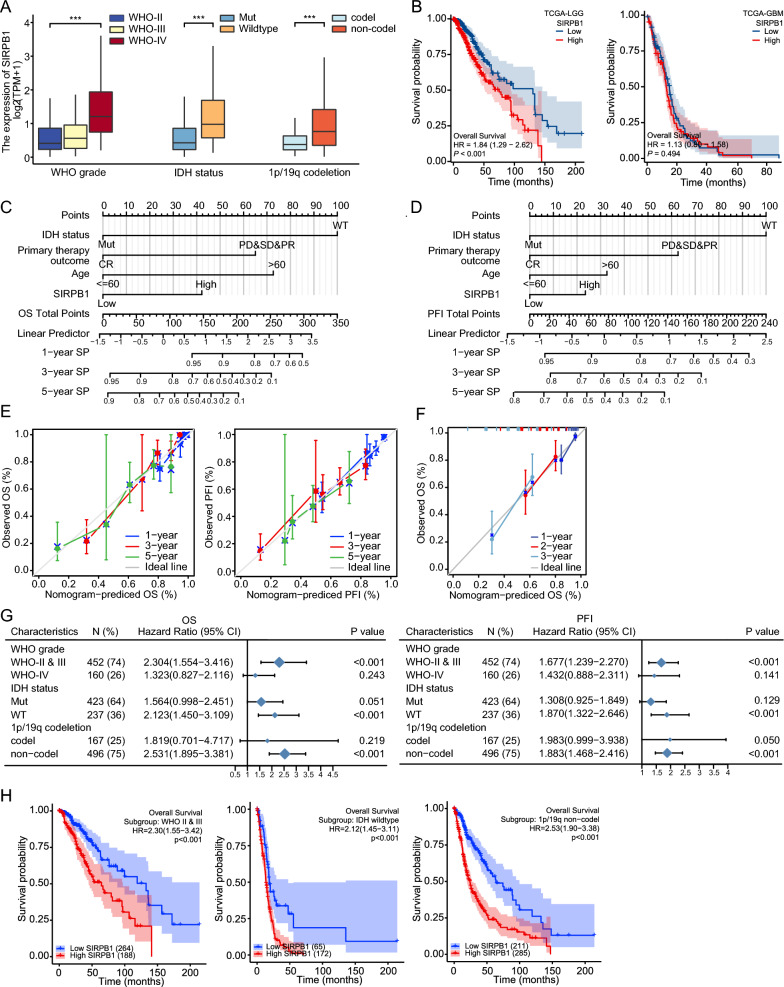
SIRPB1 in Clinicopathological Context. **A** SIRPB1 correlation with WHO grade, IDH status, and 1p19q co-deletion. **B** Kaplan–Meier curves for OS of TCGA-LGG and GBM. **C**, **D** Nomograms for 1-, 3-, 5-year OS and PFI. **E** Calibration plots for nomogram accuracy. **F** Calibration plot of glioma patients in the first Hospital of Jilin University for nomogram. **G** Forest plot of SIRPB1’s prognostic value in subgroups. **H** Kaplan–Meier curves for OS in specific glioma subgroups. ***P < 0.001

Statistical analysis of TCGA data showed significant correlations between SIRPB1 expression and clinical parameters like WHO grade, IDH status, and 1p/19q co-deletion (Fig. 8A, all *P* < 0.001). Additionally, a poor prognosis was predicted by the expression level of SIRPB1 in the LGG cohort, while this was not the case for the GBM group (Fig. 8B). Logistic regression analysis further confirmed SIRPB1's significant association with WHO grade, IDH status, 1p/19q co-deletion, primary therapy outcome, and EGFR status in glioma samples (Table 2).

**Table 2:** Logistics regression. SIRPB1 expression associated with clinical pathological characteristics

Characteristics	Total (N)	Odds Ratio (OR) in SIRPB1 expression	*P*
WHO grade (WHO-IV vs. WHO-II&III)	613	6.37 (4.14–10.08)	< 0.001
IDH status (Wildtype vs. Mutant)	661	4.45 (3.16–6.33)	< 0.001
1p/19q co-deletion (codel vs. non-codel)	664	0.27 (0.18–0.40)	< 0.001
Primary therapy outcome (CR vs. PD&SD&PR)	444	0.57 (0.37–0.87)	0.009
EGFR status (Mutant vs. Wildtype)	656	1.69 (1.03–2.81)	0.039
PIK3CA status (Mutant vs. Wildtype)	656	1.04 (0.58–1.87)	0.899

*CR* complete response, *PD* progressive disease, *PR* partial response, *SD* stable disease

### Construction of a prognostic model incorporating SIRPB1 and clinicopathological features

Table 3 details univariate and multivariate Cox regression analyses for OS. The multivariate model contained variables that were significant in the univariate analysis (*P* < 0.05). These included WHO grade, IDH status, 1p/19q co-deletion, primary therapy outcome, age, EGFR status, and SIRPB1 expression (all *P* < 0.001). Multivariate analysis identified IDH status, primary therapy outcome, age, and SIRPB1 expression as independent OS prognostic factors. Nomograms for OS and PFI were developed based on multivariate findings, displayed in Fig. 8C and D. The Concordance Index (C-index) for OS is 0.831 and for PFI is 0.738. Calibration plots in Fig. 8E validate the model's accuracy in predicting 1-, 3-, and 5-year survival rates, with bias-corrected lines closely aligning with the ideal line. We validated the prognostic model with the prognostic information of patients followed up by our medical institution and the level of SIRPB1 expression in IHC sections of patients and confirmed the effectiveness of this prognostic model again (Fig. 8F).

**Table 3:** Univariate regression and multivariate survival model of prognostic covariates in patients with glioma

Characteristics	Total (N)	Univariate analysis		Multivariate analysis	
		HR(95% CI)	*P*	HR(95% CI)	*P*
WHO grade (WHO-IV vs. WHO-II&III)	612	9.504 (7.162–12.611)	< 0.001	3.317 (0.958–11.487)	0.059
IDH status (WT vs. Mut)	660	9.850 (7.428–13.061)	< 0.001	3.907 (2.181–6.998)	< 0.001
1p/19q co-deletion (codel vs. non-codel)	663	0.216 (0.138–0.338)	< 0.001	0.736 (0.413–1.313)	0.3
Primary therapy outcome (CR vs. PD&SD&PR)	443	0.238 (0.115–0.489)	< 0.001	0.335 (0.152–0.736)	0.006
Gender (Male vs. Female)	669	1.230 (0.955–1.585)	0.109		
Age (> 60 vs. < = 60)	669	4.716 (3.609–6.161)	< 0.001	3.558 (2.115–5.984)	< 0.001
Race (White vs. Asian&Black or African American)	657	0.806 (0.492–1.321)	0.393		
EGFR status (Mut vs. WT)	655	3.628 (2.672–4.927)	< 0.001	1.606 (0.763–3.379)	0.212
PIK3CA status (Mut vs. WT)	655	1.011 (0.625–1.635)	0.966		
SIRPB1 (High vs. Low)	669	3.003 (2.294–3.932)	< 0.001	2.203 (1.415–3.429)	< 0.001

*CR* complete response, *PD* progressive disease, *PR* partial response, *SD* stable disease, *Mut* mutant, *WT* wildtype

### The role of high SIRPB1 expression in different subgroups of clinicopathological characteristics

The forest plot in Fig. 8G highlights SIRPB1’s prognostic impact across TCGA subgroups. Specifically, SIRPB1 was significant in WHO-II&III (HR: 2.304, *P* < 0.001), non-codel 1p/19q co-deletion (HR: 2.531, *P* < 0.001), and IDH wildtype (HR: 2.123, *P* < 0.001) subgroups. Elevated SIRPB1 levels correlated with worse OS and PFI in these specific patient categories, as shown in Fig. 8H and Additional file 1: Figure S2C, emphasizing the SIRPB1’s role in affecting the prognosis of glioma patients with varied pathological features.

## Discussion

Signal Regulatory Proteins (SIRPs) are immunoglobulin superfamily members with three main subtypes: SIRPα, SIRPβ, and SIRPγ [38, 39]. SIRPβ causes tyrosine phosphorylation through DAP12 or DAP10 adaptors even though it lacks a tyrosine-based signaling unit [6, 8]. Prior work by Song et al. [40] linked SIRPB1 to tumor growth in prostate cancer via the AKT pathway.

Considering the predominant expression of the SIRP family in neurons and myeloid cells [41] and the high frequency of TAMs in gliomas [5], Our attention was drawn to SIRPB1's function in TAMs. Using TCGA data, single-cell portal, and TIMER databases, we found a strong correlation between SIRPB1 and macrophages. Consistent with the notion that glioma-associated macrophages exhibit both M1 and M2 features, further cell line analyses and immunofluorescence staining verified SIRPB1 expression in monocytes and macrophages [42].

Numerous cells release a variety of mediators in the glioblastoma multiforme (GBM) microenvironment, such as chemokines and cytokines [43, 44]. These molecules activate G-protein-coupled receptors, influencing cell migration and gene expression [45, 46]. Chemokines and their receptors are pivotal in cancer growth and immune cell interactions [47, 48]. Our research connects SIRPB1 to important chemokines, cytokines, and immunological markers using GSEA and PPI network design, indicating its function in regulating immune-related pathways.

The JAK/STAT pathway is essential for various cellular functions, including immune responses and inflammation [49]. JAK is activated by extracellular chemicals, phosphorylating STAT proteins, which in turn alter the nucleus’s gene expression patterns [50]. This pathway regulates macrophage phenotype and activation. For example, interferon-γ increases inflammation and microbial death by activating STAT1 [51], while IL-4 and IL-13 activate STAT6, which is crucial for M2a macrophage polarization [52]. We looked into how macrophage polarization markers and STAT phosphorylation were affected by SIRPB1 deletion. While most classic markers remained stable, TNFα and IL10 levels decreased in the SIRPB1^KO^ group, suggesting a nuanced role for SIRPB1 in macrophage function. We propose two potential explanations for this observation. First, one or more of the molecules LPS, IFN-γ, IL-4, and IL-13 may serve as ligands for SIRPB1. However, direct interaction between these compounds and SIRPB1 has not been demonstrated by either endogenous or exogenous CO-IP assay (results not shown). Second, M1, M2, or TAMs may produce secretory factors that act as ligands for SIRPB1 during polarization, thereby activating SIRPB1 through autocrine or paracrine pathways. We intend to investigate this latter possibility in further work by employing CO-IP and protein mass spectrometry techniques to optimize the tag peptide’s location and create antibodies with increased affinity and specificity.

The literature review deepened our understanding of CCL2, IL-8, and IL1RA. CCL2, acting through pathways like PI3K/AKT and JAK/STAT, is crucial for macrophage and glioma cell migration [53–55], making it a potential target for tumor microenvironment modulation [56–58]. Numerous cells release IL-8, which attracts neutrophils and other immune cells [59–61] and is becoming more widely known for its functions in the formation of gliomas [62–64]. The natural IL1 receptor antagonist IL1RA exhibits neuroprotective and anti-inflammatory properties, and it can promote the growth of glioma by blocking the inhibitory effect of IL-1 on the proliferation of glioma cells [65–68].

Our study of a single-cell dataset using the signaling pathway of the IL1RN gene showed that IL1RN expression in glioma-associated macrophages changes from low to high. TNF, NF-κB, osteoclast differentiation, and SYK pathways may explain these findings [69]. We speculate that after SIRPB1 deletion, inadequate early SYK phosphorylation could impact downstream pathways. It will need further investigation to comprehend the intricate interactions between these routes fully.

We detected the phosphorylation level of ERK after co-culture, but we found that after about 12 h–24 h, the phosphorylation level of ERK became irregular, and even at 48h, the phosphorylation level of SIRPB1^KO^ could be higher than that of SIRPB1^WT^ in some repeated experiments, possibly due to the activation of some feedback mechanism. For example, Zhang et al. [70] found that two transcripts of IL1RN (ENST00000259206.9 and ENST00000361779.7) were generated by mutually exclusive exons, which could inhibit ERK through some negative feedback mechanism. Between macrophages and microglia in vivo and the THP-1 model in vitro, there were variations in their expression profiles.

We have made every effort to investigate the link between the key components of gliomas, including tumor cells, TAM, and blood vessels; however, the true condition in vivo cannot yet be fully restored. In an effort to investigate further SIRPB1 in vivo experimental mechanisms, our team is building *SIRPB1* knockout mice. Future studies could explore the potential of SIRPB1 as a therapeutic target through the development of specific inhibitors or monoclonal antibodies.

We have shown a negative correlation between high levels of SIRPB1 and important clinical markers such as WHO grade, IDH status, and 1p/19q co-deletion, which is the first study to investigate SIRPB1's function in the immune milieu of gliomas. Our study, for the first time, investigate SIRPB1’s role in glioma’s immune microenvironment, and associates high SIRPB1 levels with poor prognosis and key clinical markers, such as WHO grade, IDH status, and 1p/19q co-deletion. Keeping this in mind, and considering Roelands et al. [26, 27] and the results that have been published so far, we try to interpret the role of SIRPB1 in gliomas from a variety of angles, such as ICR. We found that in the samples with high ICR, the high expression of SIRPB1 still indicates a poor prognosis. It is also established that SIRPB1 may not “exist” but rather be “involved” in the pathological process of glioma in conjunction with the outcomes of in vitro investigations. We found that SIRPB1 had no prognostic significance when analyzing GBM cohorts alone, and we further analyzed the distribution of SIPRB1 expression in the whole LGG and GBM cohorts. We found that the expression of SIRPB1 in the GBM cohort was generally higher than that in the LGG cohort (Additional file 1: Figure S2B). One possible explanation for no correlation between SIRPB1 and GBM prognosis is that forced subdivision into SIRPB1-HIGH and SIRPB1-LOW in GBM with generally high expression of SIRPB1 will result in insufficient sample size of SIRPB1-LOW to produce sufficient statistical differences. Biological features, immunological microenvironment, and subtype diversity are all more complex in GBM, and while these may be important in predicting prognosis, they are not with simple GBM analysis.

## Conclusions

This work established SIRPB1’s function in the glioma inhibitory immune milieu. High expression of SIRPB1 is associated with poor prognosis. SYK affects the expression of CCL2, IL1RA, and IL8 and participates in the formation of the glioma immune microenvironment, which is considered to be an independent risk factor for glioma patients. According to our findings, SIRPB1 might be a useful prognostic indicator for gliomas.

### Supplementary Information

**Additional file 1.** **Figure S1**: The relative expression of Western blot in Figure 1, 4, 6 and 7. **Figure S2**: A. Single-Cell Analysis of CXCL8 and CCL2 Expression. B. The expression of SIRPB1 in LGG and GBM cohort. C. Kaplan-Meier curves for PFI in specific glioma subgroups. **Table S1**: Antibodies uesd for Western blot, Immunohistochemical and Immunofluorescence staining. **Table S2**: Primers used for qPCR and PCR. **Table S3**: DEGs between high and low SIRPB1 samples in TCGA-GBMLGG cohort. **Table S4**: Significant terms obtained in Enrichment analysis of DEGs between high and low SIRPB1 samples in TCGA-GBMLGG cohort. **Table S5**: Within macrophage and microglia cells, significant terms obtained in Enrichment analysis of DEGs between high and low SIRPB1 samples in single-cell dataset GSE117891.

## Data Availability

The datasets analyzed for this study can be found in the https://www.gtexportal.org/, https://xenabrowser.net/datapages/, https://www.ncbi.nlm.nih.gov/geo/query/acc.cgi?acc=GSE117891. The original data generated during the current study are available from the corresponding author on reasonable request.

## Abbreviations

SIRPB1: Signal Regulatory Protein Beta 1
TCGA: The Cancer Genome Atlas
GTEx: The Genotype-Tissue Expression
TME: Tumor microenvironment
LGG: Low grade glioma
GBM: Glioblastoma multiforme
GO: Gene ontology
BP: Biological process
CC: Cellular components
MF: Molecular function
KEGG: Kyoto encyclopedia of genes and genomes
PPI: Protein–protein interaction
GSEA: Gene set enrichment analysis
ssGSEA: Single sample gene set enrichment analysis
DEG: Differentially expressed gene
TAM: Tumor associated macrophage
