# Supplementary figures and images for: SIRPB1 regulates inflammatory factor expression in the glioma microenvironment via SYK: functional and bioinformatics insights
Source: J Transl Med. 2024 Apr 9;22:338. doi: 10.1186/s12967-024-05149-z (PMC11003053; doi:10.1186/s12967-024-05149-z)

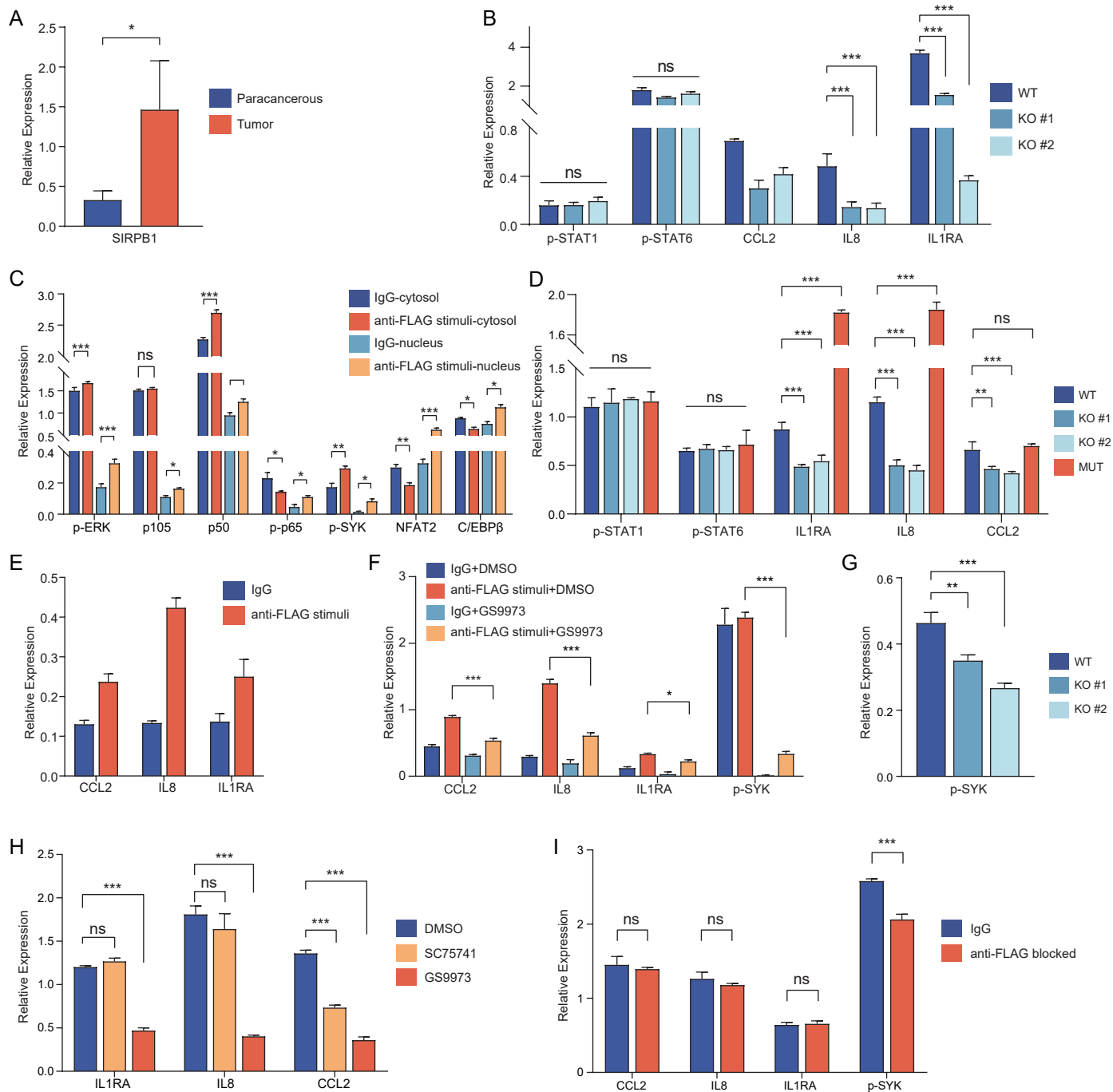

Supplement: Supplementary file 1 — Additional file 1. Figure S1: The relative expression of Western blot in Figure 1, 4, 6 and 7. Figure S2: A. Single-Cell Analysis of CXCL8 and CCL2 Expression. B. The expression of SIRPB1 in LGG and GBM cohort. C. Kaplan-Meier curves for PFI in specific glioma subgroups. Table S1: Antibodies uesd for Western blot, Immunohistochemical and Immunofluorescence staining. Table S2: Primers used for qPCR and PCR. Table S3: DEGs between high and low SIRPB1 samples in TCGA-GBMLGG cohort. Table S4: Significant terms obtained in Enrichment analysis of DEGs between high and low SIRPB1 samples in TCGA-GBMLGG cohort. Table S5: Within macrophage and microglia cells, significant terms obtained in Enrichment analysis of DEGs between high and low SIRPB1 samples in single-cell dataset GSE117891. [file 12967_2024_5149_MOESM1_ESM.zip › Additional file/Figure S1-mod.pdf]

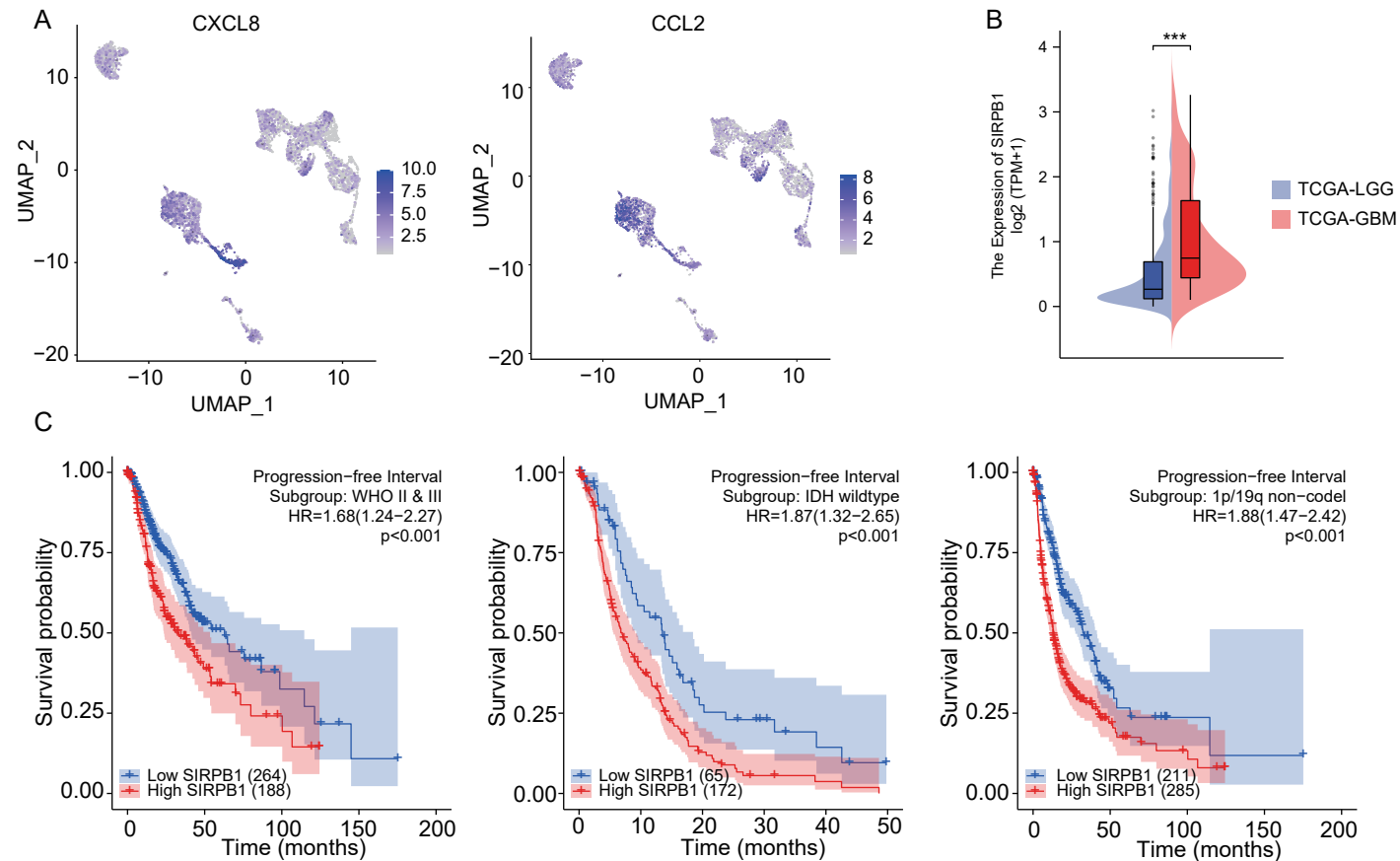

Supplement: Supplementary file 1 — Additional file 1. Figure S1: The relative expression of Western blot in Figure 1, 4, 6 and 7. Figure S2: A. Single-Cell Analysis of CXCL8 and CCL2 Expression. B. The expression of SIRPB1 in LGG and GBM cohort. C. Kaplan-Meier curves for PFI in specific glioma subgroups. Table S1: Antibodies uesd for Western blot, Immunohistochemical and Immunofluorescence staining. Table S2: Primers used for qPCR and PCR. Table S3: DEGs between high and low SIRPB1 samples in TCGA-GBMLGG cohort. Table S4: Significant terms obtained in Enrichment analysis of DEGs between high and low SIRPB1 samples in TCGA-GBMLGG cohort. Table S5: Within macrophage and microglia cells, significant terms obtained in Enrichment analysis of DEGs between high and low SIRPB1 samples in single-cell dataset GSE117891. [file 12967_2024_5149_MOESM1_ESM.zip › Additional file/Figure S2-mod.pdf]
